# Abnormal spontaneous regional white-matter brain activity in patients with obsessive-compulsive disorder

**DOI:** 10.3389/fpsyg.2025.1728241

**Published:** 2026-01-13

**Authors:** Yanan Shen, Tianyue Wang, Yongyi Ye, Shumei Li, Peiru Wu, Changhe Fan, Guihua Jiang

**Affiliations:** 1 Department of Radiology, Taizhou Hospital of Zhejiang Province, Taizhou, China; 2 The Department of Medical Imaging, The Affiliated Guangdong Second Provincial General Hospital of Jinan University, Guangzhou, China; 3 Department of Radiology, Dongguan Songshan Lake Central Hospital, Dongguan, China; 4 Department of Psychiatry, The Affiliated Guangdong Second Provincial General Hospital of Jinan University, Guangzhou, China

**Keywords:** functional magnetic resonance imaging, Neurology, obsessive-compulsive disorder, Psychiatry, white matter

## Abstract

**Introduction:**

Previous studies on white matter (WM) in patients with obsessive-compulsive disorder (OCD) have focused primarily on its structural aspects. This study aimed to investigate any abnormal spontaneous WM neural activity in patients with OCD.

**Methods:**

The study was based on resting-state functional magnetic resonance imaging (fMRI) data from 27 patients with OCD and 24 matched healthy controls (HC). Regional homogeneity (ReHo) and the amplitude of low-frequency fluctuation (ALFF) were used to explore spontaneous neural activity changes in the subjects’ WM regions. A two-sample Student’s *t*-test was performed, and correlations between the Yale-Brown Obsessive-Compulsive Scale (Y-BOCS), Hamilton Anxiety Scale (HAMA), and Hamilton Depression Scale (HAMD) scores were analyzed.

**Results:**

The ReHo in the left posterior limb of the internal capsule (LPLIC) and right superior corona radiata (RSCR) of the OCD group was significantly higher than those in the HCs (pTFCE–FWE < 0.001). The ALFFs in the right superior longitudinal fasciculus (RSLF) and right cerebral peduncle (RCP) of the OCD group, by contrast, were significantly lower than those in the HCs (pTFCE–FWE < 0.05). There was no correlation between the clinical symptoms of patients with OCD and their abnormal amplitude of low-frequency fluctuation (ALFF) and ReHo values.

**Discussion:**

Abnormal spontaneous WM activity was observed in several brain regions in patients with OCD. This activity may help explain the cognitive inflexibility often observed in this patient group.

## Introduction

Obsessive-compulsive disorder (OCD) is a prevalent mental disorder characterized by recurring and intrusive thoughts (obsessions) and/or repetitive ritualistic behaviors (compulsions). It accounts for a substantial proportion of global mental disorders. According to a recent nationally representative survey, the lifetime prevalence of OCD in China was 2.4% (Huang et al., 2019). OCD is associated with a relatively high risk of suicide, and a recent meta-analysis confirmed that at least one in 10 patients with OCD attempts suicide during their lifetime, while nearly half have suicidal ideations (Angelakis et al., 2015; Albert et al., 2019; Pellegrini et al., 2020). Patients with OCD experience substantial impairments in their quality of life and social functioning, and the World Health Organization (WHO) ranks it as one of the 10 most debilitating conditions. The specific etiology of OCD remains unclear; hence, its occurrence and developmental mechanisms merit further investigation.

Researchers have proposed a classical model of OCD that is based on its neuropsychology and the distinct neuroimaging features it presents in terms of cortico-striato-thalamo-cortical (CSTC) circuits (Saxena et al., 1998; Milad and Rauch, 2012; Van Den Heuvel et al., 2016). These circuits project from the frontal-cortical regions to the striatum, onward to the thalamic sites, and back from the thalamus to the cortex to complete the loop (Milad and Rauch, 2012; Jalal et al., 2023). CSTC circuits involve sensorimotor, cognitive, affective, and motivational processes. The gray matter (GM) in circuits such as the prefrontal cortex (PFC)—particularly the orbitofrontal cortex (OFC) and the anterior cingulate cortex (ACC)—seems to be associated with the major psychopathology of OCD (Van Den Heuvel et al., 2016; Shephard et al., 2021; Jalal et al., 2023). Although considerable progress has been made in the study of GM abnormalities in patients with OCD, much less attention has been paid to the white matter (WM) tracts that connect the brain regions implicated in this disorder. The WM lies beneath the GM cortex, comprises millions of nerve fibers that connect neurons in different brain regions into functional circuits, and is essential for impulse conduction (Fields, 2010). Exploring WM abnormalities in patients with OCD to gain a deeper understanding of their association with brain diseases is of great significance. Changes in the microstructure of the WM have been observed in patients with OCD—such as in the corpus callosum, corona radiata, anterior limb of the internal capsule, and both superior and inferior longitudinal fasciculi. However, previous studies of WM in OCD have been limited to structural changes.

Researchers have recently proposed that the blood oxygen level-dependent (BOLD) signal reflects neural activity in WM fiber tracts (Gawryluk et al., 2014; Grajauskas et al., 2019; Huang et al., 2023). In the past, the BOLD signal in WM was often handled as a nuisance covariate to be removed by regression; it was considered physiological noise with no physiological significance (Grajauskas et al., 2019; Li et al., 2020). This is typically attributed to the fact that cerebral blood flow (CBF) and cerebral blood volume (CBV) are thought to be below the threshold of detection in the WM, and the observed WM activation may be spurious because of residual effects from oxygenation changes in the GM vasculature (Fraser et al., 2012; Gawryluk et al., 2014; Li et al., 2019). However, there are two separate arterial and venous systems for the GM and WM, ensuring no vascular interactions between the different tissue types (Huang et al., 2018; Li et al., 2019, 2020). Conversely, an increasing number of researchers have shown that WM plays a role in information transmission in the central nervous system and that there are neural activities in the WM (Huang et al., 2018, 2023; Grajauskas et al., 2019; Mishra et al., 2020). BOLD signals in the WM can be used to effectively describe neuronal activity and psychopathology using multiple methods such as positron emission tomography (PET), cerebrovascular reactivity (CVR) studies after hypercapnic challenges, and resting or task-state magnetic resonance imaging (MRI) (Mazerolle et al., 2010; Thomas et al., 2014; Poublanc et al., 2015; Li et al., 2019; Guo et al., 2022). Methods of analyzing WM BOLD signals are gradually being applied in research on various brain diseases such as autism, schizophrenia, and Parkinson’s disease (Li et al., 2020; Ma L. et al., 2022; Wu et al., 2023).

In this study, we aimed to investigate whether spontaneous neural activity observed in the WM of patients with OCD was abnormal. We targeted the WM fiber tracts of patients with OCD to explore the functional abnormalities of these tracts in the resting state. We used the amplitude of low-frequency fluctuation (ALFF) and regional homogeneity (ReHo) to characterize abnormalities in spontaneous neural activity. ALFF and ReHo are commonly used methods of analyzing BOLD-functional MRI (fMRI) data, which can reflect the intensity of spontaneous neural activity and its consistency across various brain regions, respectively. This approach’s test-retest reliability and repeatability are high, and the results can be used to effectively characterize brain neural activity (Zang et al., 2004; Yu-Feng et al., 2007). Based on the results of previous studies on the abnormal WM microstructure of patients with OCD, we hypothesized that these patients may have abnormal regions in terms of WM microstructure—such as in the superior/inferior longitudinal fasciculus, corpus callosum, and anterior limb of the internal capsule.

## Materials and methods

Participants: Written informed consent was obtained from all participants. The Ethics Committee of the Guangdong No. 2 Provincial People’s Hospital approved the study. This study was carried out in accordance with the latest version of the Declaration of Helsinki.

The inclusion criteria for patients in this study were as follows: (1) The patients satisfied the criteria outlined in the Diagnostic and Statistical Manual of Mental Disorders, version 5 (DSM-V); (2) Their main symptoms were obsessive-compulsive symptoms; (3) Their ages ranged from 18 to 60 years, and both sexes were included; (4) They were right-handed; (5) They had no history of major psychiatric or neurological diseases; (6) At the time of each patient’s first psychiatric episode, there was no use of any anti-obsessive-compulsive drugs or other psychotropic drugs, or the patient was in the stable phase of drug treatment and did not receive electroconvulsive therapy. The exclusion criteria for patients with OCD included (1) serious body disease, a history of head injury, mental or neurological infection, or tumor history; (2) history of alcohol or drug abuse; (3) intense signals on conventional MRI imaging; (4) female patients who were pregnant, nursing, or menstruating; and (5) patients who had contraindications or were unable to undergo examination.

Forty-six patients with OCD were enrolled. In some instances, the data quality was poor or incomplete, owing to factors such as head motion artifacts. Ultimately, 27 patients with OCD and 24 healthy controls were included. Two or more senior physicians in our hospital’s Department of Psychology and Psychiatry diagnosed all participants.

### The Yale-Brown Obsessive-Compulsive Scale

The Yale-Brown Obsessive-Compulsive Scale (Y-BOCS), which represents the most widely used scale for diagnosing OCD, was used to assess the severity of obsessive-compulsive symptoms. It incorporates obsessive-thinking and obsessive-behavior scores, with higher scores denoting more severe symptoms. The Hamilton Depression Scale (HAMD) and Hamilton Anxiety Scale (HAMA) were used to evaluate patients with depression or anxiety.

### Magnetic resonance imaging data acquisition

MRI data were acquired using a 3.0 T Ingenia MR imager (Philips Healthcare, Best, Netherlands) at the Department of Medical Imaging, Guang Dong No. 2 Provincial People’s Hospital. During the resting-state scan, the participants wore both earplugs and headphones. They were instructed to keep their eyes closed, relax, and not think of anything specific or fall asleep. The resting state fMRI (rs-fMRI) acquisition parameters were as follows: repetition time (TR)/echo time (TE): 2,000 ms/30 ms; Flip angle: 90°; slices: 33; slice thickness: 3.5 mm; field of view (FOV): 224 mm × 224 mm; voxel size: 3.5 mm × 3.5 mm × 3.5 mm; time matrix: 64 × 61; acquisition time: 15 min.

### Data processing

All fMRI data preprocessing was conducted using the DPARSF toolbox^1^. The following steps were performed: (1) Removal of the first 10 time points to signal equilibrium and allow for participant adaptation to the scanning noise. (2) Slice time correction and exclusion of participants with a maximum motion of >2 mm or 2. (3) Re-orientation and betting, co-registration of the T1 segmented images to the functional space, and segmentation into GM, WM, and cerebrospinal fluid (CSF) using the new segmentation algorithm. (4) Regression of noise signals (CSF and head motion nuisance signals). We did not regress out WM or global signals to retain as many of the neural signals of interest as possible (Huang et al., 2018; Ma H. et al., 2022). (5) Normalization: this step differs considerably in processing WM versus GM. An individual WM mask was derived using a 90% threshold on the WM probability map obtained during the structural image segmentation (Huang et al., 2018). When the images had been resliced, and the dot product had been calculated, individual WM templates were applied to functional images to obtain new functional images containing only the WM signal. Finally, spatial normalization was performed, and smoothing, linear detrending, or filtering were selected based on the required indicators. (6) A group-level WM mask was created based on the individual-level WM images for subsequent statistical analysis. Voxels identified as WM in >90% of participants were included when generating the group-level WM. Based on the Harvard–Oxford Atlas, the subcortical regions were removed from the mask.

### Image processing and analysis

Rs-fMRI was used to assess changes in brain function. This study used ReHo and ALFF to explore changes in WM function in patients with OCD.

For the ALFF analysis, a fast Fourier transform (FFT) was then performed to transform the time sequences into a frequency series. The square root of the power spectrum was calculated to obtain the average square root of the ALFF measurement for each voxel in the 0.01–0.15 Hz range. We subtracted the average ALFF value from each voxel’s ALFF value and divided the result by the standard deviation of the whole-brain ALFF map. Finally, A Gaussian kernel (4 mm) was used to smooth the data.

For the ReHo analysis, Kendall’s coefficient of concordance (KCC) was used to calculate the similarity between a single voxel and the surrounding 26. Average ReHo values and standard deviations were then calculated. Finally, a 4 mm smoothing core was used to obtain the Z-transformed ReHo (szReHo).

### Statistical analysis

Statistical analyses were conducted using SPSS Statistics version 26.0 (IBM Corp., Armonk, NY, USA). Age-related differences were assessed using the independent two-sample Student’s *t*-test, sex differences were compared using the chi-squared test, and two-sample Student’s *t*-tests were performed to determine differences in age, education status, and total Y-BOCS score. A two-sample Student’s *t*-test was adopted for the Z-transformed ALFF (zALFF)/ smoothed and szReHo maps across the two groups. *P*-values were generated through permutation-based TFCE in randomize with 5,000 permutations^1^, adjusting for gender and age. Pearson’s correlation analysis was performed to clarify the relationships between abnormal ReHo/ALFF values in significantly different regions and to analyze the clinical characteristics of the patients with OCD in terms of Y-BOCS, HAMA, and HAMD scores. The significance threshold was set at *p* < 0.05.

## Results

### Demographic and clinical characteristics

The demographic and clinical data of the participants are shown in Table 1. The two groups did not differ significantly in terms of age or sex.

**TABLE 1:** Demographic and clinical characteristics of the study participants (mean ± standard deviation [SD]).

Parameter	Patients with OCD (*n* = 27)	HC (*n* = 24)	X^2^/*t*-value	*P*-value
Age (years)	28.37 ± 2.12	24.58 ± 1.91	−1.756	0.09
Sex (number)	16M, 11F	13M, 11F	0.134	0.714
Y-BOCS	20.78 ± 1.18	–	–	–
HAMA	19.96 ± 2.21	–	–	–
HAMD	20.96 ± 1.94	–	–	–

OCD, obsessive-compulsive disorder; HC, healthy controls; Y-BOCS, Yale-Brown Obsessive-Compulsive Scale; HAMA, Hamilton Anxiety Scale; HAMD, Hamilton Depression Scale.

### Differences between the ReHo of patients with OCD and HCs

**FIGURE 1:**
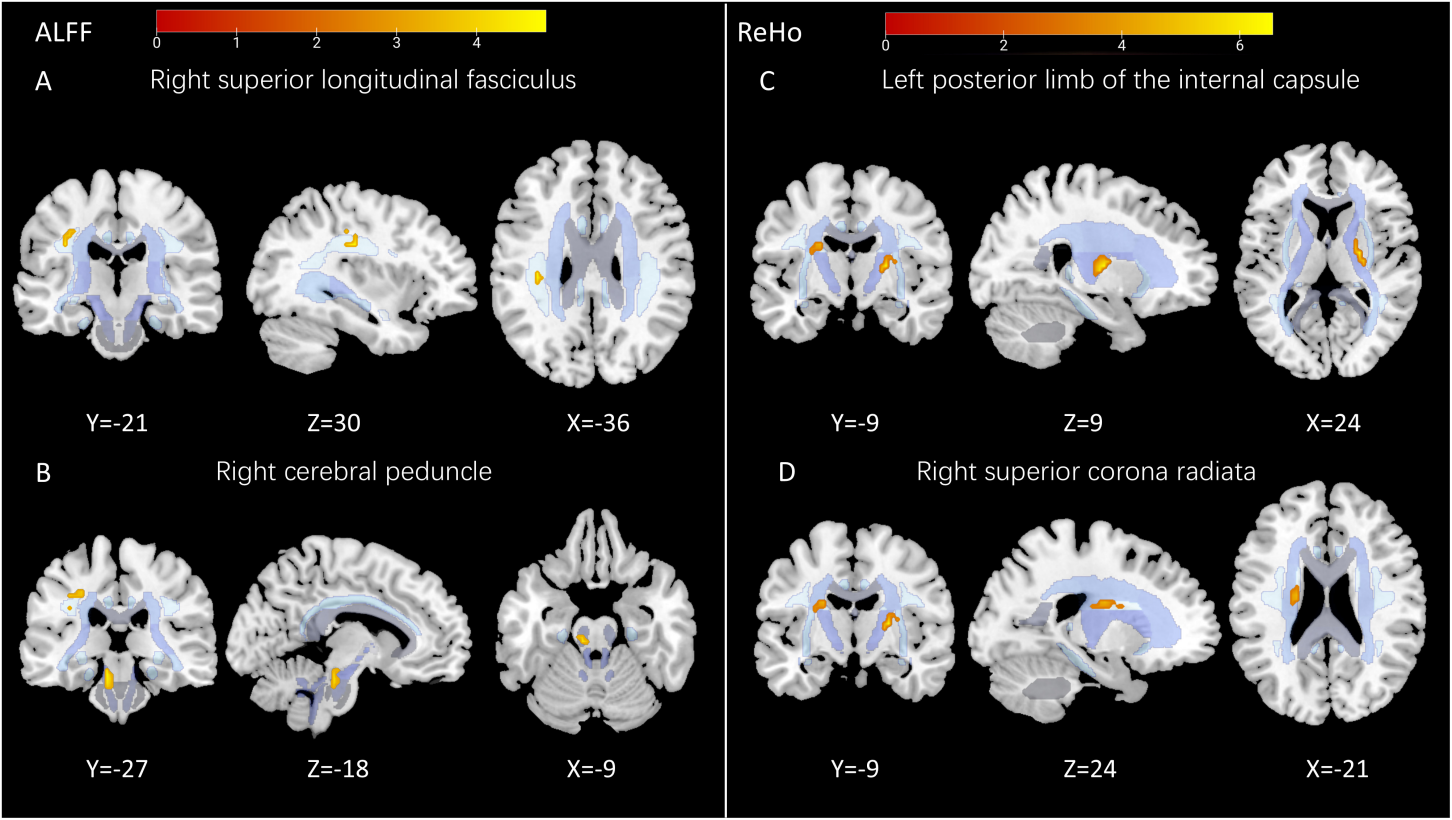
Significant differences in white matter architecture. **(A)** Patients with OCD showed higher ALLFs in the right superior longitudinal fasciculus (RSLF); **(B)** patients with OCD showed higher ALLFs in the right cerebral peduncle (RCP); **(C)** HCs showed higher ReHo in the left posterior limb of the internal capsule (LPLIC); **(D)** HCs showed higher ReHo in the right superior corona radiata (RSCR). The image overlays with the JHU atlas.

Significantly higher ReHo was observed in the left posterior limbs of the internal capsule (LPLIC) and right superior corona radiata (RSCR) of patients with OCD than in those of HCs (pTFCE–FWE < 0.001; Figure 1 and Table 2).

**TABLE 2:** Regions of WM showing abnormal ReHo, ALFFs, in patients with OCD compared with those of HCs.

Metrics	Tract (JHU-atlas)	Voxels	MNI coordinates			*T*-value
			X	y	z	
ReHo	Posterior_limb_of_internal_capsule_L	40	24	−9	9	6.56
	Superior_corona_radiata_R	38	−21	−9	24	4.92
ALFF	Cerebral_peduncle_R	17	−9	−27	−18	4.80
	Superior_longitudinal_fasciculus_R	26	−36	−21	30	4.87

MNI: Montreal Neurological Institute; JHU, Johns Hopkins University.

### Differences between the ALFFs of patients with OCD and HCs

The OCD group showed a significantly lower ALFF in the right superior longitudinal fasciculus (RSLF) and right cerebral peduncle (RCP) than did the HC one (pTFCE–FWE < 0.05; Figure 1 and Table 2).

### Correlations between alterations in abnormal regions and clinical scale scores

No significant correlations were observed between ReHo, ALFFs, and Y-BOCS scores (*p* > 0.05).

## Discussion

This study is the first to explore changes in spontaneous neural activity in the WM regions of patients with OCD, using functional WM analysis characterized by the common resting-state brain function indicators ReHo and ALFF. The combination of ReHo and ALFF can effectively reflect local neural activity in WM regions and compensate for the shortcomings of existing research on this subject. Our results show that, compared with HCs, patients with OCD had lower ALFF values in the RSLF and RCP and higher ReHo values in the LPLIC and RSCR.

We observed lower ALFFs in the RSLF of patients with OCD than in those of HCs, which suggests weakened local nerve activity in the RSLF. From an anatomical perspective, the superior longitudinal fasciculus (SLF) encompasses a heterogeneous set of bidirectional fibers that connect different regions of the frontoparietal “control” network (FPN) (Fontenelle et al., 2011; Magioncalda et al., 2016; Gan et al., 2017). Several studies have reported abnormalities in the internal connectivity of the FPN in patients with OCD, but their results are inconsistent (Göttlich et al., 2014; Gürsel et al., 2018; Liu et al., 2023). Most suggest that the FPNs of patients with OCD show within-network hypoconnectivity; however, one study that used a graph theoretical approach detected higher within-FPN connectivity in patients with OCD (Harrison et al., 2013; Göttlich et al., 2014; Gürsel et al., 2018). As an essential connecting fiber of the FPN, reduced activity of the SLF provides further evidence of low connectivity within the frontoparietal network. The frontoparietal network contains flexible hubs in the cognitive control system and coordinates attention, cognitive control, and working memory (Kim et al., 2021; Shephard et al., 2021). Several studies examining the WM structures of patients with OCD have suggested that the volume of the SLF is altered, the structural integrity of the fibers is impaired, and this correlates with the severity of obsessive-compulsive symptoms (Fontenelle et al., 2011; Jayarajan et al., 2012; Spalletta et al., 2014; Gan et al., 2017). Notably, abnormal SLF alterations were also observed in the WM. We suspect that the reduced spontaneous neural activity of the SLF in this study may be correlated with cognitive inflexibility in OCD, which leads to the maladaptive patterns of repetitive, inflexible cognition and behavior (Gruner and Pittenger, 2017; Liu et al., 2023).

Our results also showed that the ReHo values of the LPLIC and RSCR were higher in patients with OCD than in HCs, suggesting that the coordinated neuronal activity of these two fasciculi is higher in patients with OCD. A previous study on abnormal WM structural connectivity in adults with OCD reported that these patients had lower fractional anisotropy (FA) values in the left superior corona radiata (SCR) (Gan et al., 2017). The SCR carries nerve fibers from the upper cortical region, primarily connecting the parietal lobe to other brain regions. SCR damage or lesions may lead to problems with parietal lobe function. The activity in the parietal lobe may also be related to attentional set-shifting, planning, and response inhibition (Hampshire and Owen, 2005; Peng et al., 2012). One task-state fMRI study used a novel approach in which specific components of attentional shifting were fractionated and found that the posterior parietal cortex mediated changes in stimulus-response mapping (Hampshire and Owen, 2005). In another fMRI study using a task-switching paradigm, patients with OCD exhibited no significant activation in the parietal lobe during the task and significantly higher error rates in task-switching compared with HCs. Task-switching requires the ability to disengage attention from a previous task, and the examination of attention-shifting suggested parietal dysfunction in patients with OCD (Gu et al., 2007; Peng et al., 2012). Our results show that abnormal neural activity in the SCR may affect the descending transmission of parietal nerve signals, leading to attentional set-shifting impairment in patients with OCD. This is considered to reflect a lack of cognitive flexibility and may be related to the repetitive nature of OCD symptoms and behaviors (Peng et al., 2012).

We also observed significantly higher ReHo in the LPLIC in patients with OCD than in HCs, indicating greater neural coordination. The LPLIC is a crucial pathway connecting the cerebral cortex to the brainstem. The anterior region of the posterior limb contains corticospinal tract fibers. In contrast, the posterior region of the posterior limb conveys thalamic signals to sensorimotor cortical areas (Sullivan et al., 2010). Structural changes in the posterior limbs of the internal capsule have been reported in several diffusion tensor imaging studies of the WM in OCD, including lower FA values and higher MD (mean diffusivity) and RD radial diffusivity values (Fontenelle et al., 2011; Magioncalda et al., 2016). A recent cognitive task-related MRI study using a target detection task found that patients with OCD made significantly more errors during switching from negative internal thoughts to a non-affective externally oriented task, and the higher MD value in the internal capsule was correlated with the percentage of errors made, which may reflect the higher activity and connectivity in this region (Lochner et al., 2012; Magioncalda et al., 2016). Our results showed that the PLIC increases autonomic nervous activity and tends to be more consistent in OCD, which is consistent with changes in the WM microstructure of the LPLIC in patients with OCD. We speculate that abnormal neural activity in the PLIC may be linked to obsessive thoughts, as patients with OCD cannot stop negative thoughts voluntarily. However, further studies with larger cohorts are warranted to validate these findings.

## Limitations

Our study had several limitations. First, the sample size was relatively small; hence, a larger sample size is required to further validate our findings. Second, our patient cohort had a considerable prevalence of psychiatric comorbidities such as depression and anxiety, which may have affected the specificity of our findings. It must be acknowledged, however, that such comorbidities are common in patients with OCD. Therefore, excluding patients with such comorbidities would result in a narrower OCD clinical phenotype in the patient sample, which would likely not be representative of the general population of patients with OCD. Finally, no correlations were observed in the correlation analysis of this study, and the relationship between abnormal WM activity and clinical scales remains unclear. This may be because of the large number of comorbidities in our sample and variations in years of treatment between the different patients with OCD, which may have affected the heterogeneity of WM functional changes. At present, the findings are relatively limited, our next step involves utilizing white matter fiber tracts as seeds for functional connectivity analysis. This will enable a more comprehensive exploration of group differences in whole-brain connectivity patterns.

## Conclusion

To our knowledge, this study is the first to examine WM functional imaging in patients with OCD. The results showed that, in addition to structural changes, spontaneous neural activity in the WM of patients with OCD differs from that in HCs. Our study used a novel approach to provide new evidence for WM damage in patients with OCD. In addition to WM structural changes, patients with OCD have local spontaneous abnormalities in the WM, such as in the superior longitudinal fasciculus, which may affect information transmission between brain regions. These findings provide an alternative avenue for elucidating functional brain abnormalities in patients with OCD.

## Data Availability

The raw data supporting the conclusions of this article will be made available by the authors, without undue reservation.

## Footnotes

1
http://rfmri.org/DPARSF
